# Global tourist flows under the Belt and Road Initiative: A complex network analysis

**DOI:** 10.1371/journal.pone.0272964

**Published:** 2022-08-16

**Authors:** Oleksandr Shymanskyi, Jue Wang, Yue Pu

**Affiliations:** School of International Business, Southwestern University of Finance and Economics, Chengdu, Sichuan, China; University of Belgrade Faculty of Organisational Sciences: Univerzitet u Beogradu Fakultet organizacionih nauka, SERBIA

## Abstract

This study applies complex network analysis to examine global tourist flows network in the context of Belt and Road Initiative (BRI). Using tourist flows data between 221 countries/regions over 1995–2018, we investigate the nature and development patterns of structural properties of global network as well as factors influencing its formation. The descriptive analysis indicates that global tourist network was a sparse network with small world network characteristics. According to centrality characteristics, China showed the most influence in the BRI group, while Germany and the United States possessed key roles among non-BRI countries/regions. Exploratory analysis demonstrated significant influence of gravity variables in global, BRI and non-BRI tourist networks. This research advances existing tourism theory and provides practical implications for policymakers.

## Introduction

International tourism is a promising sector of trade in services that was demonstrating one of the fastest growth rates. The number of international arrivals reached 1,460 million in 2019. Although it was a smaller 4% increase compared to 7% in 2017 and 6% in 2018, the industry saw a tenth consecutive year of sustained growth in tourist arrivals. Similarly, tourism spending continued increasing, especially among the world’s top ten spenders, despite global economic slowdown. In particular, export revenues from tourism reached US $1.7 billion with China, the USA, Germany, the United Kingdom and France being among the top five world spenders [1].

Tourism industry was also placed in an important strategic position by the Belt and Road Initiative, launched by China in the end of 2013. Having cultural and historical resources to promote mutual exchanges between the countries/regions along the BRI, international tourism was expected to revive the ancient Silk Road route [2]. Since the BRI started, it gained support from over 140 countries/regions [3], some of which jointly hosted ‘Year of Tourism’ and already set up various tourism cooperation mechanisms [4]. Along with that, the Chinese government was putting efforts to facilitate tourist flows between the BRI countries/regions through visa relaxation, risk management and direct flights [5].

Indeed, international tourist flows for long time was in the focus of tourism industry players and researchers, who typically examined them through the analysis of tourist arrivals and tourism revenues in econometric methods [5–9]. Despite that, structural properties of global tourist flows received less attention in tourism research [10]. With respect to an overly increasing number of the BRI members, understanding their flow patterns in relation to other countries/regions could provide valuable insights. Additionally, identification of major determinants of global tourist flows network as well as tourism networks of BRI and non-BRI countries/regions should increase tourism management efficiency and marketing competitiveness in destinations.

A complex network analysis is a mathematic and graph theory based approach that concerns itself with structural analysis and visualization of flows, movements and relationships between network actors [11]. Having originated from social fields, the application of network analysis became interdisciplinary as scholars started experimenting with investigating various types of relationships [12]. Actors who establish relations could be individuals, organizations and other entities, whereas goods, services and information among others might represent different types of relationships [13].

In tourism literature, the network analysis approach was employed to measure tourist flows in particular destinations [14–16] and between countries [10, 17]. For example, Shih [18] applied this methodology to analyze drive tourism destinations focusing on node ties, while Leung, Wang [14] investigated movement patterns of overseas tourists in Beijing during the Olympics. By utilizing complex network, Guo, Zhang [19] examined the fluctuation patterns of monthly inbound tourist flows in China sharing valuable insights on the nature of tourism demand. Shao, Huang [10] applied this approach to illustrate the evolution of international tourist flows over 1995–2018 focusing, in particular, on properties of tourist flows network as well as the roles and functions of countries/regions within it.

Similar to network analysis, the BRI research was receiving increasing attention in tourism literature recently (see S1 Table). This is unsurprising since the B&R initiative views tourism as one of its main components that represents mutual exchanges and friendly cooperation aimed at fostering people-to-people bonds. For instance, Ahmad, Draz [20] investigated the effects of tourism on environmental situation in Chinese key BRI provinces. Deng and Hu [2] focused on the spillover effects of Chinese outbound tourism to 55 BRI countries. Huang, Han [5] demonstrated that the BRI policy had positive effect on China’s inbound tourism, while Li, Shi [4] found positive influence of BRI policy on inbound tourism and tourism revenues of participating countries. Liu and Suk [21] examined sustainable tourism development strategy between China and Azerbaijan within the BRI, whereas Li, Tavitiyaman [22] defined factors influencing tourist arrivals from Russia and Mongolia in China. Chen, Cui [23] studied the relationship between economic growth and tourism revenue along the BRI.

While tourism studies related to the BRI typically use econometric techniques, to the best of our knowledge, the network analysis was not applied to examine tourist flows across the BRI countries. Further, a considerable number of tourism publications that uses this methodology commonly focuses on structural characteristics (e.g. centrality characteristics) of networks [10, 11, 16], which are purely of descriptive nature. Agreeing with Liu, Huang [15], we state that descriptive studies are not able to reveal the underlying mechanisms of network formation. In addition, a number of investigated tourism networks are static [17, 24] and do not provide enough information to examine how structural characteristics evolved over time. Although more sophisticated methods of network analysis such as dynamic networks [10, 25], the Quadratic Assignment Procedure (QAP) regression [15, 26] and agent-based network model [27] are being used, their overall number is relatively low. In addition, gravity variables that emphasize the impact of various dimensions of distance [28–31], commonly used in tourism demand modeling [32], were not investigated in network setting.

Based on the mentioned above, our research employs the complex network analysis methodology to analyze tourism data of 221 countries/regions over 1995–2018 in the context of BRI. Specifically, descriptive part examines the nature of global tourist flows network and centrality properties in the most influential BRI and non-BRI countries/regions, whereas exploratory part, using distance related gravity variables [28, 32], applies the QAP approach to analyze and compare the influencing factors in global, BRI and non-BRI tourist networks. As such, current study contributes to literature in a number of ways. Firstly, we describe the network centrality characteristics focusing on major countries/regions that belong to BRI and non-BRI groups with further comparative analysis. Secondly, our research employs longitudinal data to demonstrate the evolution of travel patterns of visitors from BRI and non-BRI countries. Thirdly, this study applies a more sophisticated exploratory analysis that is based on gravity theory to investigate underlying factors of network formation in global, BRI and non-BRI tourism networks.

The remainder of this paper is organized as follows. Section 2 describes data sources and network analysis methodology. Section 3 discusses the results obtained from descriptive and exploratory parts of network analysis. Section 4 sums up the results with subsequent theoretical and practical implications as well as limitations and future research directions.

## Materials and methods

### Data sources and preparation

#### Dependent variable

The data on tourist flows between countries/regions were accessed from United Nations World Tourism Organization [33]. It provides tourism data from eight information sources that are based on countries’ methods to report their tourism statistics. Since countries employ varying reporting techniques, we had to select the information source individually for every country that would provide the largest amount of tourism data. Although the tourism data were available from 1995 to 2019 year, the last year was excluded due to a large share of missing data that could have negatively influenced the interpretation of network structural features. As such, the study period of current research is from 1995 to 2018 years.

#### Independent variables

The data on independent variables were collected from the gravity database available at CEPII [34] website. They include *physical distance* between most populated city of each country/region, *common language*, *contiguity*, *common religion* that adds up products of shares of Catholics, Protestants and Muslims in a particular pair of countries/regions, gross domestic product (in current thousands US dollars) used for calculating *economic distance* expressed as absolute difference between GDPs of certain countries/regions.

#### Belt and Road counties

In this paper, the information on countries/regions participating in the B&R Initiative (see S2 Table) was kindly provided by Green Belt and Road Initiative Center [3]. Following Kang, Peng [35], we regarded Hong Kong (China) and Macau (China) as non-BRI members, while Taiwan (China) was labeled as ‘other’ country/region within non-BRI group owing to its economic development and international classification [33].

### The methodology of network analysis

**Fig 1:**
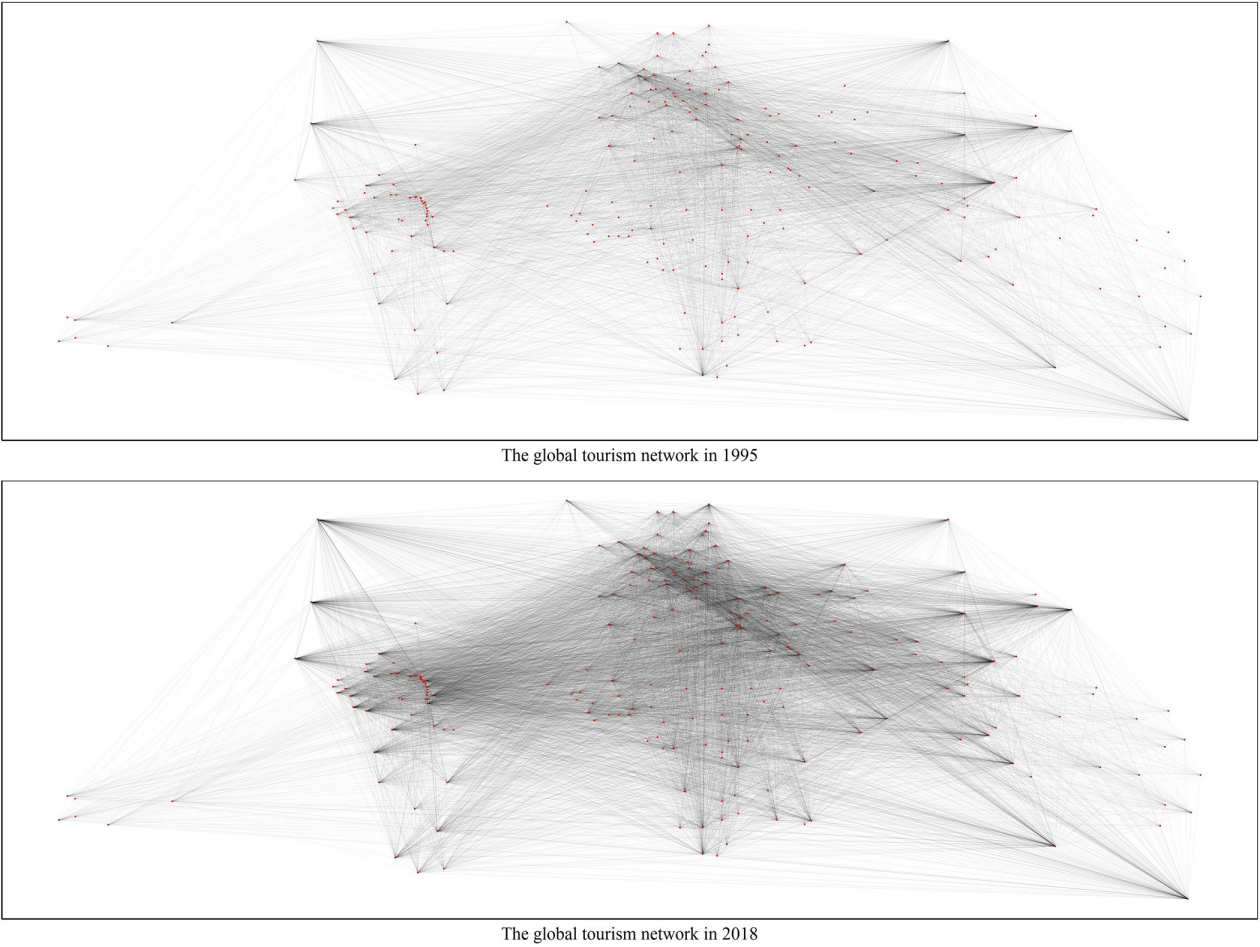
The development of global tourist flows network.

In this research, we used network analysis to examine global tourist flows. Graphically, simple representation of our network is illustrated in Fig 1, in which importance of international tourist flows is reflected through the thickness of ties. Using tourism data of 221 countries/regions over 1995–2018, we analyze network indicators that are discussed below.

#### Number of nodes and ties within network

The node number represents the quantity of countries/regions our network contains in a particular year and the number of ties demonstrates how many outbound or inbound tourist flows occur between countries/regions in a certain year.

#### Network density

The network density represents how close node relationship is. It is expressed as the ratio of present ties in relation to total possible ties in network of *N* nodes. Network density was expressed by Wasserman and Faust [36] as:

$$D=\frac{T}{\left[N(N-1)\right]}$$ (1)

in which *D*–represents network density; *T*–means number of ties; *N–*denotes number of nodes;

#### Transitivity

Transitivity is a network concept demonstrating the probability of country/region A and country/region B that both have links to country/region C to have ties between each other. According to Barrat, Barthelemy [37], transitivity can be expressed as enumeration of the proportion of node triples that form triangles:

$$c_{i}^{w}=\frac{1}{s_{i}(k_{i}-1)}\sum_{j,h}\frac{w_{ij}+w_{ij}}{2}a_{ij}a_{ih}a_{jh}$$ (2)

in which *c*_*i*_^*w*^ represents the weight of two ties of node *i* in every triplet formed in neighborhood of the node *i*;

*s*_*i*_ *(k*_*i*_*− 1)* is the normalization factor that considers the weight of each tie; *w*_*ij*_ and *w*_*ih*_ denote the weights of ties; and *a*_*ij*_, *a*_*ih*_, *a*_*jh*_ imply nodes of the adjacency matrix.

#### Reciprocity

Reciprocity demonstrates the ratio of ‘mutuality’ connections between countries/regions in a directed network. According to Newman, Forrest [38], it has the following mathematical formulation:

$$r=\frac{L^{↔}}{L}$$ (3)

in which *r* stands for the ratio of the number of ties that point to both directions *L*^*↔*^ to the total number of ties *L*.

#### Average shortest path length (APL)

In our network, the APL would imply links between country/region *i* and country/region *j*. Mathematical formulation of average path length was provided in the study by West [39]:

$$APL=\frac{1}{\frac{1}{2}N(N+1)}\sum_{i\geq j}d_{ij}$$ (4)

in which *APL*–stands for average shortest path length; *N*–denotes number of nodes; *d*_*ij*_−means distance between countries/regions *i* and *j*.

#### Betweenness centrality

This indicator demonstrates the ‘bridging’ role of a node in network based on shortest paths. Freeman [40] gave the following mathematical expression of betweenness centrality:

$$C_{B}(v)=\sum_{s\neq t\neq v\in V}\frac{\sigma (s,t|v)}{\sigma (s,t)}$$ (5)

in which *σ (s*, *t | v)* stands for the total number of shortest paths *s* and *t* through *v*; and *σ (s*, *t)* represent the total number of shortest paths *s* and *t* regardless of passing through *v*. In case shortest path is unique, *c*_*B*_ *(v)* only counts the shortest paths through *v*.

#### Degree centrality

Degree centrality represents the number of node’s ties demonstrating its ability to connect directly to other nodes in a network. Following studies on social networks [41, 42], out- and in-degree centralities can be expressed through following mathematical expressions:

$$k_{i}^{out}=\sum_{j=1}^{N}a_{ij},k_{i}^{in}=\sum_{j=1}^{N}a_{ji}$$ (6)

in which *k*_*i*_^*ou*t^ and *k*_*i*_^*in*^ represent out- and in-degree centralities of a country/region, respectively; *a*_*ij*_ and *a*_*ji*_ are elements in the respective unweighted adjacent matrices.

#### Strength centrality

Strength centrality also express how close the connection between nodes is, and ascribes weights to ties between nodes. Barrat, Barthelemy [37] provided following mathematical formulation of out- and in-strength centralities:

$$s_{i}^{out}=\sum_{j=1}^{N}w_{ij},s_{i}^{in}=\sum_{j=1}^{N}w_{ji}$$ (7)

in which *s*_*i*_^*out*^ and *s*_*i*_^*in*^ denote out- and in-strength centralities of a country/region, respectively; *w*_*ij*_ and *w*_*ji*_ are the weights of ties between nodes *i* and *j* or *j* and *i*.

### The quadratic assignment procedure

The QAP approach is employed to examine the relationship between the dependent variable and a number of independent variables. By using this technique, we can obtain significance levels of independent variables as well as pseudo R^2^ –which is quite similar to results that ordinary least squares (OLS) estimation produces and solves the problem of autocorrelation [15]. Mathematically, the QAP regression has the following expression:

$$A_{y}=\beta_{0}A_{1}+\beta_{1}A_{x1}+\beta_{2}A_{x2}+\beta_{3}A_{x3}+\ldots +Z$$ (8)

in which *A*_*y*_ is an adjacency matrix that denotes dependent adjacency matrix; *A*_*1*_ is an *n × n* matrix of 1’s; *A*_*xi*_ represents the *ith* independent adjacency matrix; and *Z* denotes *n × n* matrix of independent normal variables with 0 mean and variance *σ*^*2*^.

In this study, tourist flows between countries/regions represent dependent variable, a weighted matrix ranging from 215 × 215 countries/regions in 1995 to 221 × 221 countries/regions in 2018. In this matrices, each cell (row *i*, column *j*) contains a number of tourists from origin country/region *i* to destination country/region *j*. Further, a number of matrices of independent variables such as physical distance, common language, contiguity, common religion and economic distance are selected in this study. We expect that all variables expect physical distance will have positive influence on tourist flows.

## Results and discussion

### The structural features of global tourist flow network

Analysis of the network characteristics allows us to understand better how tourist flows were evolving in global network and what development patterns emerged. Table 1 demonstrates structural features of 221 countries/regions within global tourism network over 1995–2018 years.

**Table 1:** The structural features of the global tourist flow network over 1995–2018.

	1995	2000	2005	2008	2010	2012	2014	2016	2018
**No. Nodes**	215	216	220	219	220	221	221	221	221
**No. Ties**	7015	10334	11210	12416	13924	15112	15435	16232	15888
**Density**	0.152	0.223	0.233	0.26	0.289	0.311	0.317	0.334	0.327
**Transitivity**	0.513	0.576	0.592	0.617	0.647	0.67	0.671	0.69	0.682
**Reciprocity**	0.33	0.419	0.432	0.457	0.472	0.488	0.497	0.507	0.492
**APL**	1.898	1.745	1.737	1.704	1.688	1.66	1.649	1.634	1.61

The number of nodes, in general, was constantly growing from 215 in 1995 to 221 in 2018. Considering the number of ties, we can observe positive trend up to 2016 interrupted by slight decline. We believe that the number of tourist flows (ties) between countries/regions, in fact, was consistently increasing until the end of the study period. The same logic applies to three following properties–density, transitivity and reciprocity, which demonstrated positive trends despite lower values in the end of the study period. With that in mind, density indicated increased interaction among countries/regions in tourist flows network possibly due to globalization [43] and tourism related government policies such as the BRI [4]. At the same time, relatively low value of density implied that global tourist flows network was a sparse network and exhibited scale-free power-law distribution [44]. Further, the values of transitivity reported that countries/regions had tendency to cluster together within network forming communities that were highly connected, and network reciprocity reported growing mutual cooperation between countries/regions. Finally yet importantly, the APL was continuously declining over 1995–2018 meaning that the effectiveness of tourist flows improved owing to shorter distance between countries/regions.

### The nature of global tourism network

**Fig 2:**
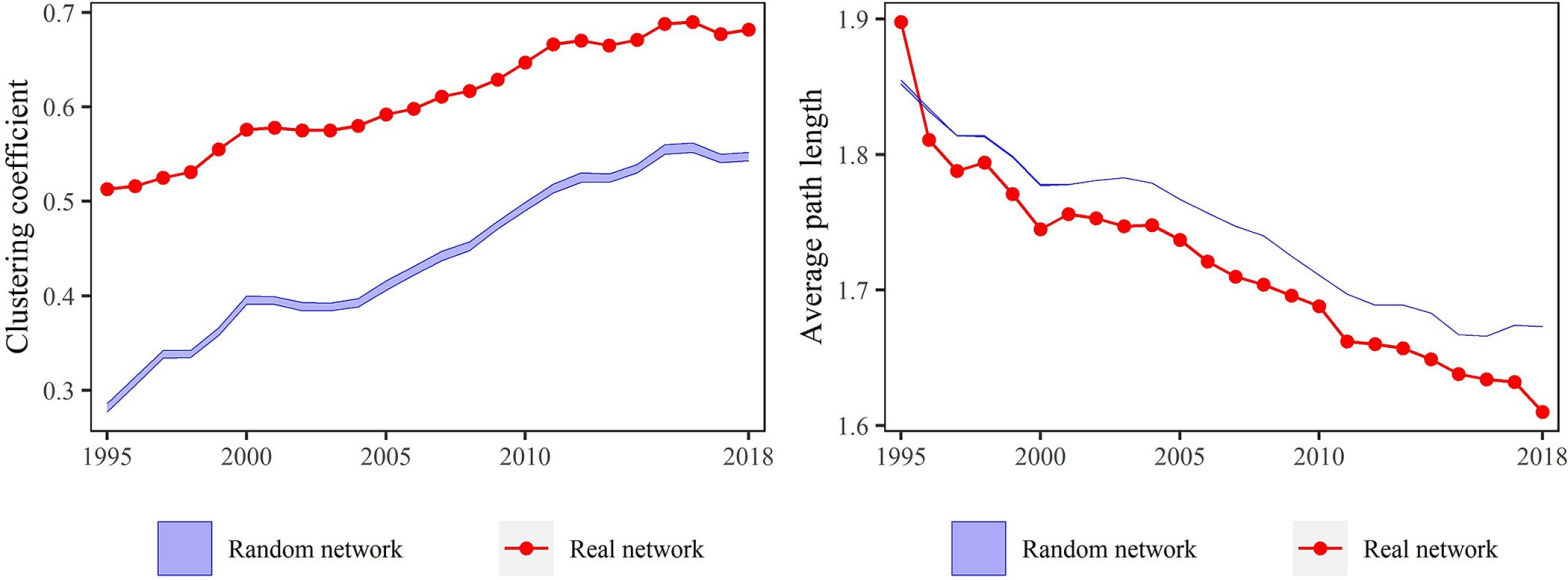
The transitivity (clustering coefficients) and average path length (APL) of the global tourist flows network. The red line represents transitivity (clustering coefficients) and average path length (APL) of real network, whereas the blue shaded area shows the maximum and minimum values of these indices obtained from 1000 random networks.

We additionally examined the transitivity (clustering coefficients) and APL of tourist flows network, values of which were subsequently compared with those of 1000 random networks that had the same number of nodes and ties to test the properties of ‘small world’ network [45]. This type of network is characterized by short mean distance between pairs of network nodes in comparison to the total number of nodes. Fig 2 illustrates that our tourism network had higher values of the transitivity (clustering coefficients) compared to those of the corresponding random network implying significant effect of tourist flows between countries/regions. Considering the APL, we can observe that their values were lower (shorter) in the real network, except for the first year. This infers that since 1996 our tourism network was demonstrating properties of small world network that are distinguished by two major characteristics: a short APL and high transitivity (clustering coefficients). This means that the actors in our network needed a rather small number of connections to reach each other [45].

### The structural features of countries/regions within the global tourist flow network

#### Mediating roles of countries/regions in global tourist flows network

Betweenness centrality shares insights regarding countries/regions that exercise power through bridging positions within the global tourism network. Table 2 reports the betweenness centralities of top 15 BRI non-BRI countries/regions for the selected years demonstrating steady downward trajectory for the majority of countries/regions. This implies that the intermediary roles of countries/regions was diminishing throughout the entire study period.

**Table 2:** Centrality properties of BRI and non-BRI groups.

	BRI						Non-BRI				
No.	1995	2005	2010	2015	2018	No.	1995	2005	2010	2015	2018
	*BETWENNESS*						*BETWENNESS*				
**1**	NZL(2594.01)	NZL(1027.37)	RUS(941.61)	CHN(1144.98)	CHN(986.81)	**1**	BEL(4261.1)	USA(3206.6)	CAN(2748.95)	USA(2502.86)	USA(2286.87)
**2**	ZAF(1842.92)	ZAF(1008)	NZL(883.98)	NZL(901.42)	NZL(815.45)	**2**	USA(2685.38)	CAN(3095.98)	USA(2744.75)	CAN(2182.39)	CAN(2174.04)
**3**	RUS(1380.49)	POL(929.23)	CHN(835.67)	RUS(772.61)	KOR(589.32)	**3**	ISR(1661.27)	ITA(2043.97)	AUS(1639.12)	AUS(1397.23)	BEL(1278.67)
**4**	CHN(1210.12)	RUS(866.52)	ZAF(812.85)	KOR(701.83)	ZAF(552.91)	**4**	HKG(965.7)	AUS(1954.3)	ITA(1451.33)	BEL(1332.75)	AUS(1153.16)
**5**	KOR(1090.05)	CHN(691.3)	KOR(773.49)	ZAF(650.26)	TUR(525.03)	**5**	GBR(780.07)	BEL(1474.95)	BEL(1187.77)	JPN(1276.17)	JPN(1002.66)
**6**	EGY(627.84)	KOR(681.47)	POL(563.01)	TUR(563.94)	POL(519.3)	**6**	CAN(762.03)	FIN(818.31)	FIN(836.43)	ITA(1206.67)	ITA(929.01)
**7**	GUY(538.91)	THA(515.31)	THA(451.44)	SAU(522.49)	SAU(401.79)	**7**	JPN(732.56)	ISR(791.06)	MEX(802.01)	FIN(793.27)	FIN(753.81)
**8**	ROU(476.68)	CHL(322.28)	SUR(393.52)	POL(512.61)	ARE(323.82)	**8**	AUS(719.27)	IND(446.57)	ISR(734.54)	ISR(624.99)	MEX(574.48)
**9**	ZMB(321.61)	ROU(294.96)	UKR(382.7)	MYS(311.82)	MYS(309.76)	**9**	DEU(382.9)	MUS(444.62)	IND(675.03)	IND(550.56)	ISR(557.62)
**10**	LBN(306.18)	EGY(209.05)	KWT(374.16)	UKR(293.33)	BGR(295.1)	**10**	BRA(278.46)	HKG(368.2)	GBR(457.45)	MEX(544.67)	IND(528.05)
**11**	CHL(279.07)	ECU(205.39)	ROU(339.15)	ROU(260.54)	ROU(281.71)	**11**	IND(264.53)	BHS(356.16)	FRA(449.71)	BRA(344.07)	HKG(276.55)
**12**	NGA(266.73)	KWT(201.6)	CHL(261.27)	UGA(257.44)	IDN(278.77)	**12**	CHE(261.61)	FRA(345.92)	JPN(366.42)	MUS(288.69)	MUS(272.17)
**13**	MAR(253.54)	SAU(200.61)	ECU(245.23)	SUR(251.04)	UKR(278.18)	**13**	BRB(244.88)	JPN(327.05)	MUS(330.76)	HKG(281.95)	BHS(260.22)
**14**	POL(169.31)	GIN(185.68)	EGY(236.56)	KWT(245.24)	ECU(203.39)	**14**	ITA(237.46)	GBR(256.36)	HKG(322.71)	FRA(277.29)	COL(235.88)
**15**	UKR(164.13)	UKR(174.4)	PAK(235.27)	TZA(233.61)	ZWE(159.9)	**15**	FRA(229.4)	BRB(255.98)	COL(319.44)	GBR(265.06)	GBR(234.8)
	*DEGREE*						*DEGREE*				
**1**	NZL(252)	POL(295)	RUS(322)	CHN(354)	CHN(346)	**1**	BEL(294)	CAN(361)	CAN(379)	USA(393)	USA(381)
**2**	ZAF(243)	RUS(288)	KOR(314)	RUS(335)	NZL(332)	**2**	ISR(245)	USA(361)	USA(375)	CAN(383)	CAN(380)
**3**	RUS(237)	ZAF(282)	ZAF(303)	KOR(330)	TUR(327)	**3**	HKG(234)	AUS(331)	AUS(346)	BEL(364)	BEL(358)
**4**	KOR(226)	NZL(274)	CHN(299)	TUR(330)	KOR(318)	**4**	USA(228)	BEL(319)	BEL(333)	AUS(362)	AUS(352)
**5**	CHN(225)	THA(272)	THA(291)	NZL(322)	POL(311)	**5**	GBR(184)	ITA(318)	ITA(329)	JPN(355)	JPN(342)
**6**	EGY(197)	CHN(268)	POL(287)	ZAF(316)	ZAF(310)	**6**	JPN(180)	FIN(277)	FIN(315)	FIN(340)	FIN(338)
**7**	ROU(192)	KOR(266)	UKR(285)	POL(310)	ARE(301)	**7**	CAN(175)	ISR(275)	IND(306)	ITA(338)	ITA(320)
**8**	MAR(187)	ROU(240)	NZL(284)	UKR(291)	IDN(294)	**8**	AUS(165)	IND(261)	MEX(305)	MEX(316)	MEX(317)
**9**	LBN(183)	KWT(235)	ECU(265)	MYS(284)	MYS(292)	**9**	DEU(164)	HKG(249)	ISR(293)	IND(314)	IND(309)
**10**	UKR(158)	LBN(230)	ROU(265)	UGA(279)	UKR(292)	**10**	ITA(155)	MUS(230)	COL(276)	ISR(298)	COL(291)
**11**	TZA(157)	SAU(224)	KWT(260)	ECU(276)	ECU(290)	**11**	MAC(154)	BHS(214)	HKG(263)	COL(289)	ISR(286)
**12**	GUY(150)	ECU(221)	EGY(259)	ROU(275)	BGR(289)	**12**	CHE(151)	JPN(205)	BHS(238)	HKG(273)	HKG(275)
**13**	NGA(148)	MAR(220)	CRI(257)	CRI(269)	ROU(287)	**13**	IND(146)	GBR(202)	MUS(232)	MUS(265)	MUS(270)
**14**	MDV(147)	UKR(218)	LBN(255)	PAN(269)	KWT(268)	**14**	CUB(143)	BRB(201)	JPN(231)	BRB(259)	JOR(260)
**15**	IRN(142)	EGY(217)	PAK(252)	SAU(269)	CRI(267)	**15**	FRA(142)	CHE(193)	GBR(231)	NIC(258)	MLI(256)
	*STRENGTH*						*STRENGTH*				
**1**	POL(84553256)	CHN(151506419)	CHN(185715519)	CHN(252073395)	CHN(304245207.52)	**1**	DEU(111161410)	USA(124480364)	USA(134880797)	USA(173869326.4)	USA(198772519.3)
**2**	CHN(50902266)	POL(74391223)	POL(68450825)	POL(91079289)	POL(101363186.42)	**2**	USA(102186507)	DEU(121385804)	HKG(126044879)	HKG(151429260)	HKG(158563877)
**3**	AUT(21283220)	RUS(39985018)	RUS(50486452)	RUS(56095242)	RUS(57072137.44)	**3**	ITA(57440100)	HKG(101516362)	DEU(115149854.15)	DEU(135336648)	DEU(153900413.28)
**4**	CZE(19349201)	UKR(30657564)	UKR(34849683)	TUR(43029410)	THA(48612352)	**4**	FRA(55507497)	FRA(88043755)	ITA(93455758.41)	FRA(103395575)	ITA(115981806.63)
**5**	RUS(15391902)	AUT(25765842)	TUR(33477800)	THA(38761520)	KOR(48388760.69)	**5**	GBR(48738782)	GBR(83588126)	FRA(92735567.07)	ITA(102198396)	FRA(110681993.13)
**6**	GRC(11386734)	TUR(23813360)	AUT(30443358)	UKR(37031243)	TUR(48101559.52)	**6**	HKG(48432895)	ITA(78832491)	GBR(80238636)	GBR(97218298)	GBR(107515544.62)
**7**	UKR(10800288)	KOR(17178406)	KOR(22977851)	MYS(36897751)	UKR(41418505.81)	**7**	CAN(35289383)	MAC(44975837)	ESP(53201709.95)	ESP(65005687)	ESP(78433787.76)
**8**	SGP(9675601)	GRC(17103801)	THA(21320545)	KOR(36651069)	MYS(39971041)	**8**	MEX(27931486)	ESP(44516086)	MAC(48993035)	MAC(54762800)	MEX(63813015.77)
**9**	TUR(9111791)	CZE(16995695)	CZE(19392885)	SGP(34727324)	AUT(37949220.52)	**9**	JPN(25808935)	CAN(41029090)	CAN(46644219)	MEX(53403063.4)	MAC(62239514)
**10**	THA(8568025)	THA(14520896)	GRC(17835771)	AUT(33858333)	SGP(36351688)	**10**	ESP(23175760)	MEX(36513781)	MEX(36981182)	CAN(52194351)	CAN(58939744.57)
**11**	KOR(8114086)	SGP(13207279)	SGP(17148478)	SAU(33232041)	SAU(36037214.54)	**11**	CHE(23018232)	JPN(29914818)	CHE(34655676.59)	JPN(42539787)	JPN(56208993.02)
**12**	ROU(7580621)	SAU(10920517)	EGY(17035371)	GRC(27246830)	GRC(32237094.69)	**12**	NLD(19229197)	NLD(29406829)	NLD(32043590.15)	CHE(39589295)	CHE(41728581.38)
**13**	ZAF(6071041)	ZAF(10390320)	ZAF(16568696)	CZE(25270773)	CZE(29866984.14)	**13**	MAC(15895637)	CHE(25924071)	JPN(31351134)	NLD(34950613)	NLD(39758968)
**14**	IDN(5919107)	EGY(10197052)	SAU(15462683)	ZAF(19596449)	VNM(28183298)	**14**	TWN(9286298)	BEL(15715815)	BEL(17867633)	TWN(26904552)	TWN(31088672.5)
**15**	SVK(5722248)	BGR(9483253)	ROU(12812353)	IDN(19131909)	IDN(26902065)	**15**	BEL(8800937)	TWN(14541831)	TWN(17831711)	AUS(22930211)	IND(29531570.08)

Centralities are sorted in descending order

**Fig 3:**
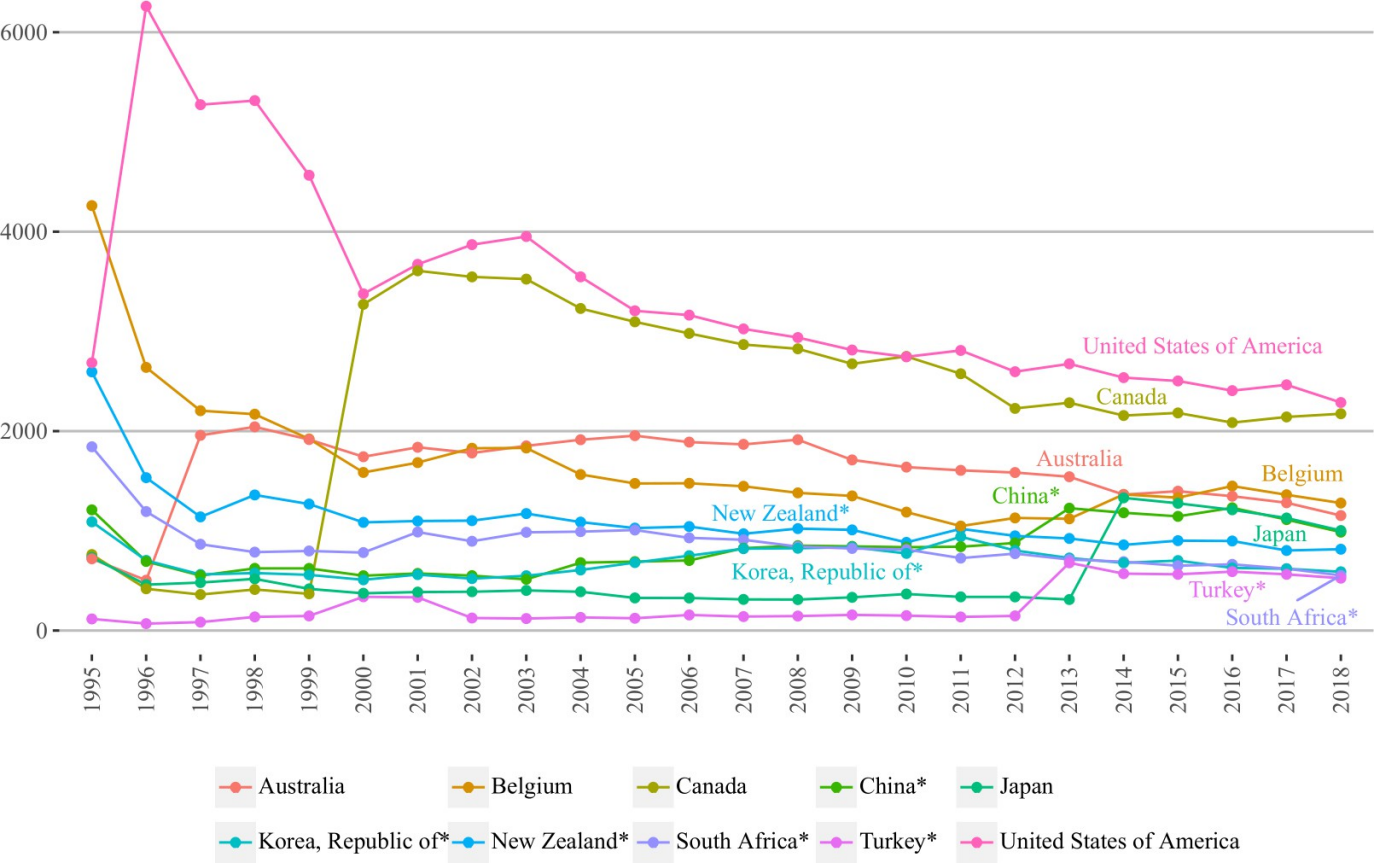
Betweenness centrality over 1995–2018 of top five BRI and non-BRI countries/regions. Countries/regions were selected based on the highest values of 2018 year; BRI marked with ‘*’.

Fig 3 illustrates the development trends of betweenness centrality for top five BRI and non-BRI countries/regions with some degree of variation over 1995–2000. In addition to clear division between both groups, all non-BRI countries/regions were located above BRI. More specifically, the United States and Canada had the highest values ranking first and second during the majority of the study period. A considerable gap existed between them and the other countries/regions (both BRI and non-BRI), whose positions were not far from each other. The remaining non-BRI countries/regions were represented by Belgium, Australia and Japan with respective third, fourth and fifth rankings. Considering the BRI members, China exceeded other BRI countries competing with Japan for fifth place over the last years, however, ranked sixth by the end of the study period. Further, New Zealand was placed right between China and three other BRI countries over 2013–2018 ranking seventh. The rest of the BRI countries/regions including Korea (Republic of), South Africa and Turkey all were having rather similar values of betweenness centrality in the last years of the study period and ranked eighth, ninth and tenth, respectively.

#### The connectedness of countries/regions in global tourist flows network

Degree centrality of countries/regions was examined to describe their connectedness with each other. In Table 2, we can see that the number of connections, in general, was increasing among BRI and non-BRI groups, however, slightly declined in the end of the study period (possibly due to slightly larger number of missing data).

**Fig 4:**
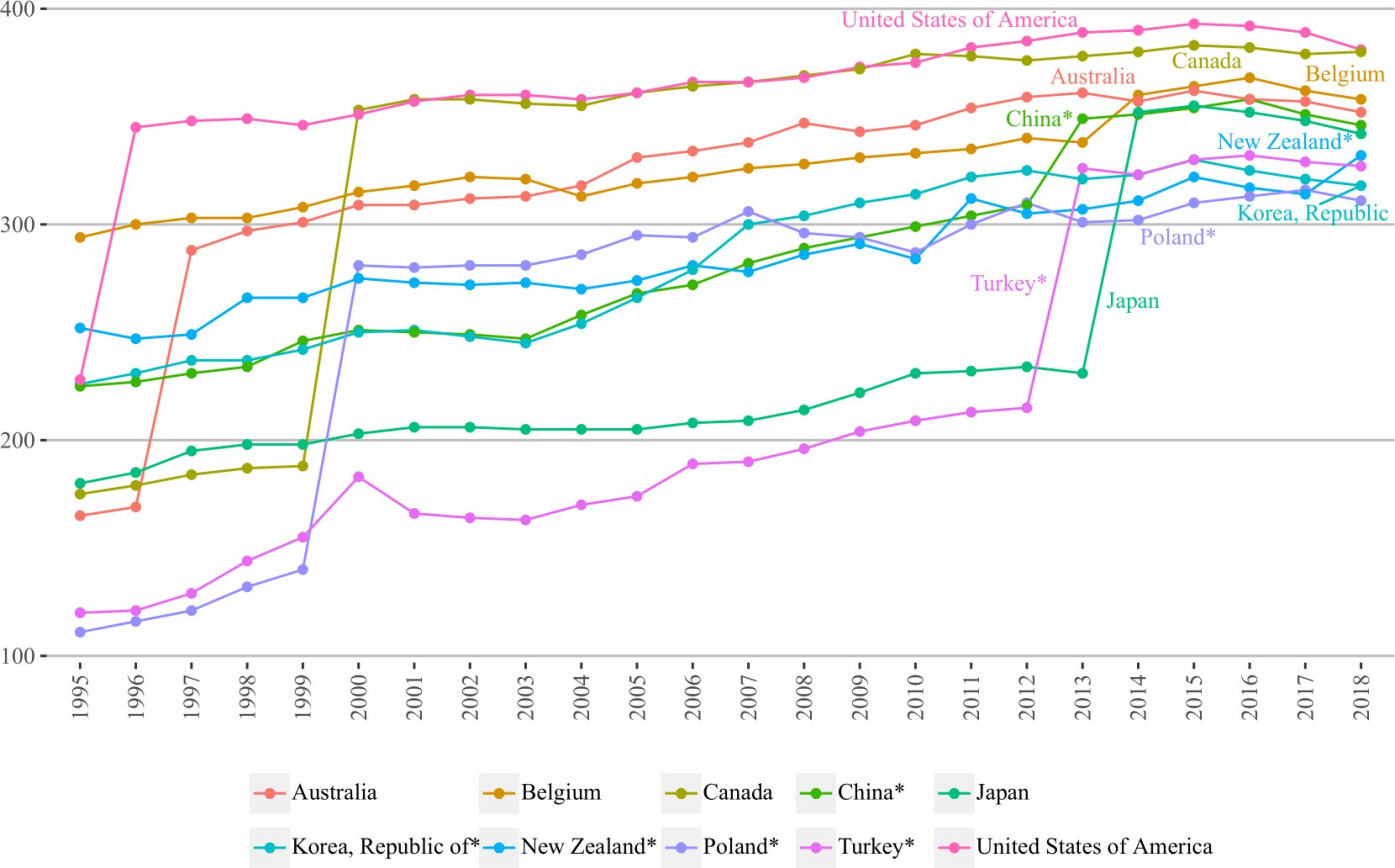
Degree centralities of top five BRI and non-BRI countries/regions over 1995–2018. Countries are selected based on the highest values of 2018 years; BRI marked with ‘*’.

Focusing on top 5 BRI and non-BRI countries/regions, Fig 4 demonstrates that non-BRI countries/regions remained the same as those in Fig 3, whereas in BRI group South Africa was replaced by Poland. The major difference with betweenness centrality is that all countries/regions (regardless of group division) had upward trend, which slightly declined in 2017 and 2018. Further, the countries’/regions’ rankings had slight changes: despite the fact that the United States, Canada, Belgium and Australia held first four rankings, now China ranked fifth having surpassed Japan (ranked sixth). The remaining four BRI countries, same as in betweenness centrality graph, were located below their non-BRI counterparts and China. Interestingly, a significant boost in degree centrality of the United States, Australia, Canada and Poland can be seen in the early years of the study period (1995–2000), while Turkey and Japan experienced rapid growth during the last years of the study period (2012–2014).

#### The impact of countries/regions in global tourist flows network

Table 2 provides the information on strength centrality of countries/regions that shows their roles on both outbound and inbound tourism markets. Opposite to betweenness and rather similar to degree centrality, the values of strength centrality were showing steady growth for the majority of countries/regions.

**Fig 5:**
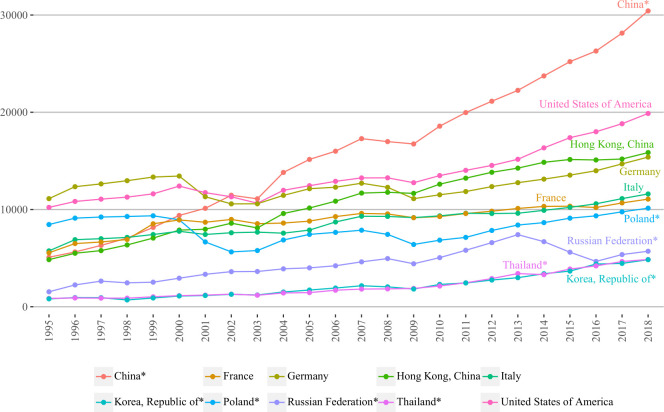
Strength centralities of top five BRI and non-BRI countries/regions over 1995–2018. In 10 thousand people, countries/regions are selected based on the highest values of 2018 years; BRI marked with ‘*’.

Five BRI and non-BRI countries/regions with the highest strength centrality values are demonstrated in Fig 5. As can be seen, this time the majority of countries/regions again had upward trend throughout the entire study period, regardless of some minor declines over 2000–2003 and 2007–2009. In particular, having exceeded all other countries/regions, China was ranking first since 2002 until the end of the study period. It is worth mentioning that the gap between China, non-BRI and the remaining BRI countries/regions was increasing after China’s accelerated growth in 2009. In non-BRI group, the United States, Hong Kong (China), Germany, Italy and France had somewhat similar development patterns with respective second, third, fourth, fifth and sixth rankings. Speaking of BRI countries/regions, we can observe quite different development trends. For instance, after 1999–2002 decline, Poland dropped from third to seventh position yielding to non-BRI group, which it was trying to catch up with ever since. In the last years of the study period, Poland got closer to France and Italy. Further, regardless of decline during 2013–2016, Russian Federation did not change its position and ranked eighth in 2018. Two remaining BRI countries/regions Korea (Republic of) and Thailand had almost the same growth trajectory repeatedly exchanging their rankings throughout the entire study period. As of 2018, Thailand ranked ninth, whereas Korea (Republic of) was tenth.

#### The impact of countries/regions on outbound tourism

We investigated out-strength centrality to define what countries/regions had significant impact in outbound tourism market. Table 3 reports out-strength of 25 BRI and non-BRI countries/regions for selected years that are of major importance for outbound tourism. In 2018, both groups accounted for 72% of the sum of out-strength centrality in the 221 countries/regions. Specifically, the share of 25 BRI made 22.3%, whereas 25 non-BRI contributed to 49.7%, respectively.

**Table 3:** Out-strength centralities of BRI and non-BRI groups.

	BRI						Non-BRI				
No.	1995	2005	2010	2015	2018	No.	1995	2005	2010	2015	2018
**1**	CZE(16179945)	CHN(31259430)	CHN(52009157)	CHN(118255520)	CHN(145796483.52)	**1**	DEU(100009657)	DEU(104281736)	DEU(93013994.15)	DEU(106774243)	DEU(122456869.28)
**2**	AUT(9666510)	RUS(18393168)	RUS(28975497)	RUS(26351010)	RUS(33718863.44)	**2**	USA(59283872)	HKG(78158331)	HKG(90016111)	USA(96106571.4)	USA(119039128.3)
**3**	UKR(7616202)	UKR(13044125)	AUT(14837967)	UKR(24609112)	KOR(33295907.69)	**3**	HKG(38237862)	USA(75346788)	USA(74904880)	HKG(92122077)	HKG(93416847)
**4**	RUS(5176449)	AUT(11818175)	KOR(14501054)	KOR(23691793)	UKR(27321943.81)	**4**	GBR(26066782)	GBR(55102126)	GBR(50328501)	GBR(61025114)	GBR(67958053.62)
**5**	BLR(4918554)	KOR(11448863)	UKR(13665689)	SGP(19952105)	SAU(20724425.54)	**5**	FRA(23803312)	FRA(32365356)	FRA(38789742.07)	FRA(44668415)	FRA(49372036.13)
**6**	SVK(4875197)	CZE(11027474)	CZE(13540756)	CZE(17161926)	CZE(19950204.14)	**6**	JPN(22494339)	MAC(26273298)	CAN(30449142)	CAN(34245787)	CAN(37820567.57)
**7**	CHN(4794003)	POL(9901041)	POL(10450021)	SAU(15272734)	SGP(18466055)	**7**	CAN(18708383)	ITA(24379952)	CHE(26412322.59)	CHE(30549158)	CHE(31683373.38)
**8**	KOR(4699070)	HUN(6344028)	SVK(8558279)	AUT(15014843)	AUT(16840438.52)	**8**	CHE(16882099)	JPN(23240220)	ITA(26324799.41)	ITA(28282419)	ITA(30635135.63)
**9**	TUR(3543743)	BLR(5956035)	MYS(8454800)	POL(13474140)	POL(15533610.42)	**9**	NLD(15618397)	CAN(22281375)	NLD(24090590.15)	NLD(24329613)	ESP(27092873)
**10**	MYS(3394476)	MDA(5270891)	BLR(7056367)	MYS(11193160)	MYS(14143285)	**10**	ITA(14384524)	NLD(22095429)	MAC(24030557)	MAC(24053986)	NLD(26604968)
**11**	SGP(2617349)	MYS(5078394)	TUR(6796365)	THA(9952605)	VNM(13293841)	**11**	ESP(8635590)	CHE(19120636)	JPN(22786058)	ESP(22812967)	MAC(26442875)
**12**	POL(2434048)	SVK(4882628)	MDA(6534821)	KAZ(9901351)	MMR(13266584)	**12**	MEX(8514126)	ESP(17127178)	ESP(20430350.95)	JPN(22805547)	JPN(25018525.02)
**13**	ROU(2388345)	SGP(4384563)	IRN(5839711)	IDN(9571493)	THA(11826162)	**13**	MAC(8311844)	MEX(14402363)	MEX(15332471)	MEX(21148220)	MEX(22116511.77)
**14**	HUN(1991305)	TUR(3789607)	SGP(5615530)	SVK(9208471)	IDN(11798836)	**14**	TWN(7060209)	TWN(11577401)	TWN(12536678)	TWN(16568867)	TWN(20177176.5)
**15**	GRC(1951107)	IDN(3559206)	ROU(5551628)	TUR(8815027)	SVK(10221190.69)	**15**	BEL(4638758)	BEL(10344177)	BEL(12073737)	AUS(15485901)	IND(19000371.08)
**16**	IDN(1883251)	PRT(3556626)	HUN(5470457)	MDA(7408842)	ROU(9873814.3)	**16**	SWE(4272744)	AUS(7905859)	AUS(11761401)	BEL(14022626)	AUS(17306093.05)
**17**	MDA(1763946)	GRC(3382278)	ZAF(5412598)	ROU(7030464)	KAZ(9765913)	**17**	ARG(3980070)	SWE(7461051)	SWE(8153805.34)	IND(12636021)	BEL(15489451.82)
**18**	THA(1744232)	THA(3039416)	THA(5399081)	BLR(6941111)	TUR(9509759.52)	**18**	AUS(3926037)	IRL(5491661)	IND(7561150)	SWE(10189170)	ARG(12714758.89)
**19**	LTU(1694772)	BGR(3027202)	IDN(5072082)	VNM(6722166)	UZB(9151879)	**19**	DNK(3433569)	DNK(5071673)	BRA(6199839)	ARG(9853964)	SWE(10645017.71)
**20**	SAU(1594043)	LTU(2959016)	SAU(4622888)	BGR(6659537)	BGR(8880674.02)	**20**	IRL(2869298)	FIN(4444328)	DNK(5904048)	BRA(9163988)	BRA(10426521.55)
**21**	ZAF(1540629)	ZAF(2941206)	PRT(4524504)	PHL(6274652)	BLR(8490578)	**21**	FIN(2788435)	NOR(4161356)	ARG(5836133)	DNK(7234409)	DNK(8005890.9)
**22**	EGY(1487729)	PHL(2919282)	KAZ(4436620)	HUN(5983725)	PHL(8217345.83)	**22**	BRA(2516202)	IND(4060921)	IRL(5578759)	IRL(6377373)	IRL(7601422.68)
**23**	PHL(1483770)	SAU(2894445)	LTU(4357206)	ZAF(5660877)	MDA(8009869)	**23**	ISR(2078387)	ARG(3997564)	NOR(5273654)	NOR(6315983)	ISR(6510844.29)
**24**	PRT(1473074)	KAZ(2885720)	GRC(4233793)	PRT(5297687)	ZAF(6570986.27)	**24**	NOR(1775795)	SYR(2951844)	FIN(5065295)	FIN(6001685)	NOR(6000194.07)
**25**	BGR(1419928)	ROU(2496762)	PHL(4216808)	MKD(4477240)	HUN(6175142.84)	**25**	IND(1605325)	ISR(2897332)	SYR(4184738)	ISR(4392757)	FIN(5392709.99)

Centralities are sorted in descending order

**Fig 6:**
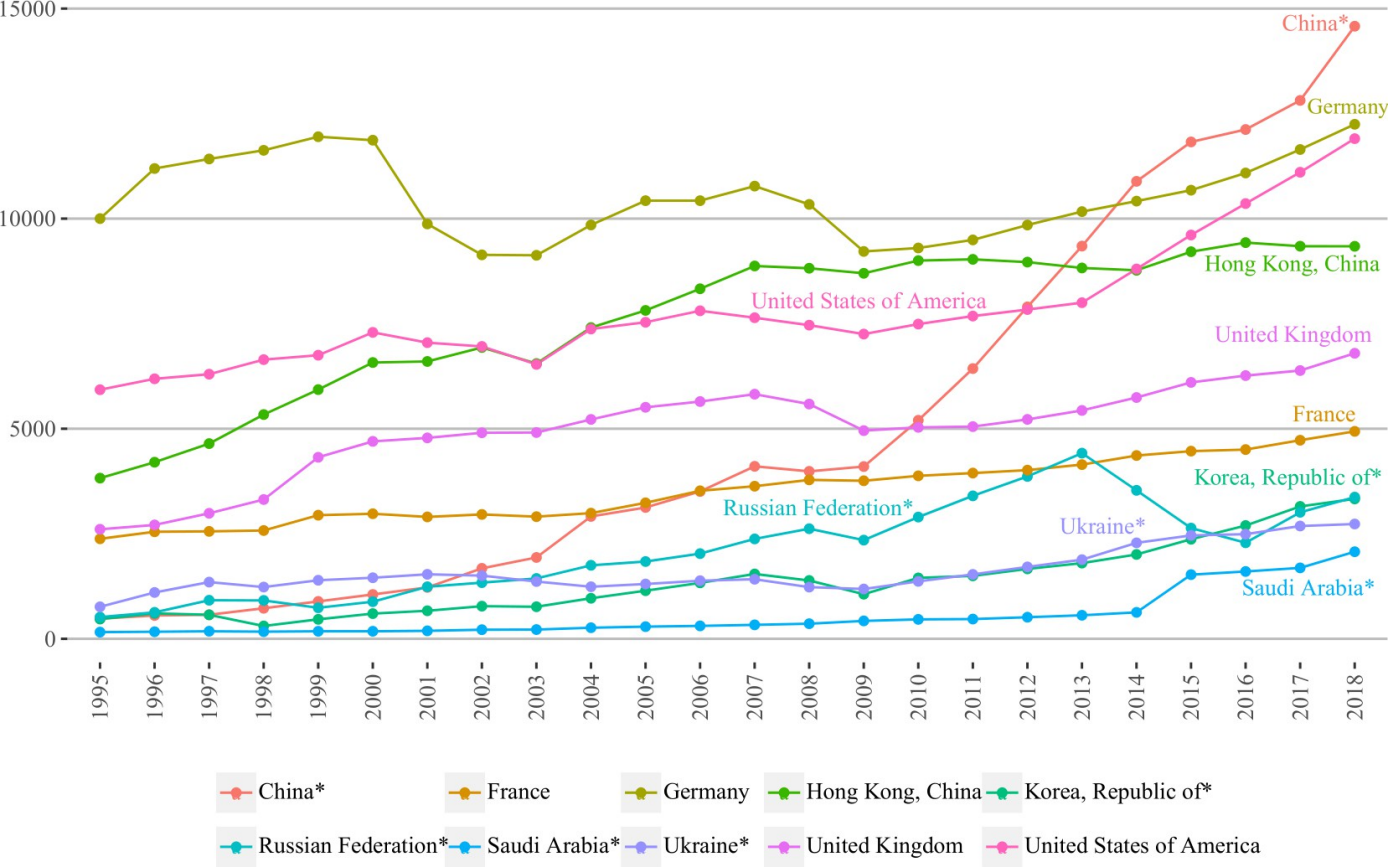
Out-strength centralities of top five BRI and non-BRI countries/regions over 1995–2018. In 10 thousand people; countries/regions are selected based on the highest values of 2018 years; BRI marked with ‘*’.

Fig 6 provides the visual representation of the out-strength centrality of top 5 BRI and non-BRI countries/regions. In 2018, China had the highest out-strength compared to other countries/regions, which is not surprising, considering country’s unprecedented economic growth and overly increasing role in outbound tourism market [46]. Indeed, China’s out-strength values saw dramatic increase from 4794003 in 1995 to 145796484 in 2018, which is more than 30 times during 24 years. Over 1995–2007, a number of countries/regions such as Saudi Arabia, Ukraine, Korea (Republic of), Russian Federation, and France were surpassed by China. Throughout the entire study period, China’s out-strength just slightly declined in 2008. Starting from 2009, it was growing at an even faster pace exceeding the United Kingdom in 2010, the United States in 2012, Hong Kong (China) in 2013 and Germany in 2014, ranking first and becoming a major generating country of outbound tourist flows until the end of the study period.

During 1995–2013, Germany was the largest source of outbound tourists and after yielding leadership to China in 2014 ranked second, being followed by the USA. Despite both Germany and the USA experienced declines in out-strength (1999–2003 and 2007–2009 in Germany, 2000–2003 and 2006–2009 in the USA), downturns in Germany were much more severe (see 2001 and 2009), whereas the USA was able to recover relatively faster and had increased its growth pace starting from 2013 and almost caught up with Germany in 2018. Hong Kong (China), in general, was growing steadily without serious effects caused by declines. Over 2003–2014, it surpassed the USA, however, quickly yielded after that period and was ranking fourth until 2018. Next, the United Kingdom and France both demonstrated rather steady growth over 1995–2018, whereas the former had somewhat steeper upward/downward trajectories.

Russian Federation was demonstrating steady growth over 1995–2013 with two slight declines in 1999 and 2009, respectively. After that, it experienced a sharp downturn during 2013–2016 due to a number of reasons that might be explained by oil price decrease, tense political relations with Ukraine [47] and subsequent sanctions imposed by Western countries [48]. Although the out-strength value was recovering since, Russian Federation did not catch up with the value of 2013 year ranking sixth by the end of the study period. By contrast, Korea (Republic of), was steadily growing over the study period without negative consequences from declines of 1998 and 2009. In 2016, it exceeded Russian Federation, but in spite of this, two years later was surpassed by the latter and ranked seventh. Over 1995–2009, Ukraine experienced smooth upward and downwards trends and had been growing steadily since 2009. This is quite surprising, considering that in the end of 2013 a series of protests took place in Ukraine, which were followed by government overthrow in 2014 and military conflict with the most severe phase during 2014–2016. None of these factors, seemingly, impeded the growth of out-strength of Ukraine, which ranked ninth in 2018. Lastly, Saudi Arabia was continuously growing during the entire study period, especially in 2015 when country’s outbound tourism market rose almost twice compared to 2014. Regardless of this, the values of Saudi Arabia were lower in relation to other countries/regions making it rank tenth in 2018.

By comparing two groups of top 5 BRI and non-BRI countries/regions, we can see that China was the only BRI country/region that had higher out-strength value than non-BRI countries over relatively long time. Another case of BRI country/region surpassing non-BRI happened in 2013, when Russian Federation exceeded France, however it quickly conceded in the next year (due to the reasons described in the paragraph above). In our opinion, non-BRI countries/regions showed higher values than the majority of BRI countries/regions since all of them were enjoying high-economic development level for a relatively long time, whereas most of the BRI countries/regions are regarded as developing or became developed rather recently. In addition, the out-strength values of the BRI countries/regions, except for China, are normally more concentrated next to each other, especially in the first and last years of the study period.

#### The impact of countries/regions on inbound tourism

In-strength centrality was analyzed to understand which countries had great influence in the inbound tourism network. Table 4 provides information on the in-strength centralities for selected years of top 25 BRI and non-BRI countries for selected years. As of 2018, both groups contributed 71.3% out of sum of in-strength centrality in the 221 countries/regions. More precisely, BRI members accounted for 34.6%, whereas non-BRI countries/regions had 36.7% share.

**Table 4:** In-strength centralities of BRI and non-BRI groups.

	BRI						Non-BRI				
No.	1995	2005	2010	2015	2018	No.	1995	2005	2010	2015	2018
**1**	POL(82119208)	CHN(120246989)	CHN(133706362)	CHN(133817875)	CHN(158448724)	**1**	ITA(43055576)	FRA(55678399)	ITA(67130959)	USA(77762755)	ITA(85346671)
**2**	CHN(46108263)	POL(64490182)	POL(58000804)	POL(77605149)	POL(85829576)	**2**	USA(42902635)	ITA(54452539)	USA(59975917)	ITA(73915977)	USA(79733391)
**3**	AUT(11616710)	RUS(21591850)	TUR(26681435)	TUR(34214383)	TUR(38591800)	**3**	FRA(31704185)	USA(49133576)	FRA(53945825)	HKG(59307183)	HKG(65147030)
**4**	RUS(10215453)	TUR(20023753)	RUS(21510955)	RUS(29744232)	THA(36786190)	**4**	GBR(22672000)	GBR(28486000)	HKG(36028768)	FRA(58727160)	FRA(61309957)
**5**	GRC(9435627)	UKR(17613439)	UKR(21183994)	THA(28808915)	GRC(27180498)	**5**	MEX(19417360)	ESP(27388908)	ESP(32771359)	ESP(42192720)	ESP(51340914.76)
**6**	SGP(7058252)	AUT(13947667)	THA(15921464)	MYS(25704591)	MYS(25827756)	**6**	CAN(16581000)	HKG(23358031)	GBR(29910135)	GBR(36193184)	MEX(41696504)
**7**	THA(6823793)	GRC(13721523)	AUT(15605391)	GRC(23084955)	RUS(23353274)	**7**	ESP(14540170)	MEX(22111418)	MAC(24962478)	MEX(32254843.4)	GBR(39557491)
**8**	TUR(5568048)	THA(11481480)	EGY(14691051)	AUT(18843490)	AUT(21108782)	**8**	DEU(11151753)	CAN(18747715)	DEU(22135860)	MAC(30708814)	MAC(35796639)
**9**	ROU(5192276)	SGP(8822716)	GRC(13601978)	SAU(17959307)	ARE(18389340)	**9**	HKG(10195033)	MAC(18702539)	MEX(21648711)	DEU(28562405)	DEU(31443544)
**10**	ZAF(4530412)	EGY(8166096)	SGP(11532948)	SGP(14775219)	SGP(17885633)	**10**	MAC(7583793)	DEU(17104068)	CAN(16195077)	JPN(19734240)	JPN(31190468)
**11**	BGR(4123063)	SAU(8026072)	ZAF(11156098)	ZAF(13935572)	SAU(15312789)	**11**	CHE(6136133)	NLD(7311400)	SYR(9297327)	CAN(17948564)	CAN(21119177)
**12**	IDN(4035856)	ZAF(7449114)	SAU(10839795)	KOR(12959276)	IDN(15103229)	**12**	IRL(4456000)	IRL(6966000)	JPN(8565076)	NLD(10621000)	NLD(13154000)
**13**	TUN(3947831)	BGR(6456051)	KOR(8476797)	UKR(12422131)	KOR(15092853)	**13**	BEL(4162179)	CHE(6803435)	CHE(8243354)	TWN(10335685)	TWN(10911496)
**14**	PRT(3879351)	TUN(6263262)	BGR(8268828)	IDN(9560416)	ZAF(14988122)	**14**	AUS(3678500)	JPN(6674598)	NLD(7953000)	CHE(9040137)	IND(10531199)
**15**	KOR(3415016)	CZE(5968221)	ROU(7260725)	EGY(9301038)	VNM(14889457)	**15**	NLD(3610800)	AUS(5489727)	IRL(5965000)	IRL(8582000)	CHE(10045208)
**16**	UKR(3184086)	KOR(5729543)	TUN(6785108)	BGR(9280916)	UKR(14096562)	**16**	JPN(3314596)	BEL(5371638)	JOR(5853473)	IND(8007624)	IRL(9695000)
**17**	CZE(3169256)	ROU(5689946)	IDN(6489884)	PRT(9023414)	BGR(12295297)	**17**	JOR(3259898)	BRA(5215313)	BEL(5793896)	AUS(7444310)	AUS(9242050)
**18**	EGY(3110408)	PRT(5146128)	NGA(6024531)	ROU(8991132)	PRT(11616095)	**18**	NOR(2621676)	SYR(4811413)	AUS(5785340)	BEL(6520679)	ARG(6911877)
**19**	HUN(1902148)	IDN(4719087)	PRT(5863227)	BHR(8511763)	EGY(11273308)	**19**	PRI(2339807)	JOR(4345709)	IND(5760469)	BRA(6274727)	BEL(6720066)
**20**	URY(1827715)	KAZ(4361703)	CZE(5852129)	CZE(8108847)	ROU(11194666)	**20**	TWN(2226089)	IND(3881747)	TWN(5295033)	DOM(4822830)	BRA(6599214)
**21**	PHL(1589841)	HRV(3654578)	KWT(5173396)	VNM(7436560)	BHR(10737689)	**21**	ISR(2171744)	DOM(3073006)	BRA(5053238)	ARG(4684839)	DOM(5587938)
**22**	MAR(1521649)	KWT(3469811)	MAR(4905616)	KWT(6901851)	CZE(9916780)	**22**	SYR(2148354)	TWN(2964430)	ARG(4526125)	PER(3445656)	PER(4417941)
**23**	CHL(1500391)	VNM(3198900)	VNM(4787900)	KAZ(6419399)	KAZ(8777152)	**23**	IND(2056539)	PRI(2944904)	DOM(3499477)	SWE(3380649)	ISR(4110138)
**24**	KWT(1432734)	MAR(3042199)	KAZ(4081213)	HRV(5149903)	KWT(8470152)	**24**	BRA(1937974)	CYP(2457501)	SWE(2890573)	NOR(3204946)	CYP(3875627)
**25**	NZL(1371115)	HUN(2814415)	HRV(3933262)	MAR(5149258)	IRN(7210649)	**25**	ARG(1709499)	SWE(2335694)	ISR(2788679)	PRI(3185889)	SWE(3844058)

Centralities are sorted in descending order

**Fig 7:**
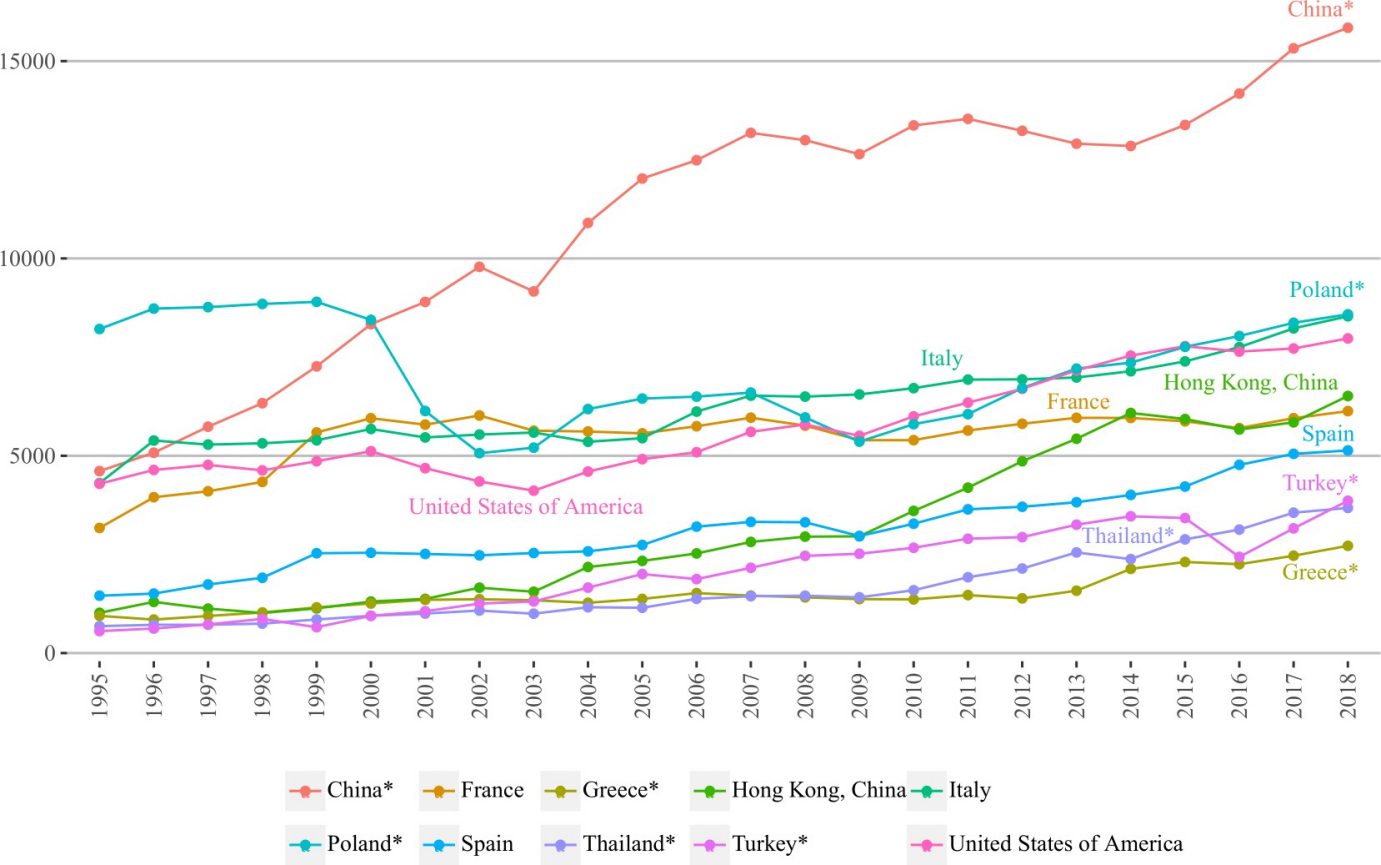
In-strength centralities of top five BRI and non-BRI countries/regions over 1995–2018. In 10 thousand people; countries/regions are selected based on the highest values of 2018 years; BRI marked with ‘*’.

In Fig 7, we can observe that the variation of in-strength centrality is lower compared to that of out-strength (Fig 6). Regarding the countries/regions, China again demonstrated dramatic growth, especially in the early years (1995–2002) of the study period. In 2001, it already ranked first holding leadership until the end of the study period, regardless of two slight declines in 2003 and during 2007–2009. The in-strength value demonstrates that China been of great interest to inbound tourists since the very beginning, whereas China’s out-strength shows that it became a major source of outbound tourists after 2009. Poland, another BRI member, experienced a series of downward/upward trends that had been decreasing/increasing the country’s ranking throughout the study period. After the second slight downturn over 2007–2009, it had been constantly growing since, having obtained the second ranking, and almost reached its highest in-strength value, which Poland once had prior to the first sharp decline (1999–2002).

As to the other leading countries/regions, Italy ranked third in 2018, which is the highest position among non-BRI countries/regions. The country’s growth can be described, in general, as positive and steady with several minor declines that were always recovered in each following year. Italy would often yield/regain its ranking mainly competing with countries/regions such as France, the United States and Poland. In 2016, it exceeded the United States (leaving it fourth ranking), and almost caught up with Poland during the last years. Quite interestingly, Hong Kong (China) had the lowest in-degree value among non-BRI countries, however, was able to surpass Spain and France due to a rapid growth over 2009–2014 and eventually ranked fifth in 2018. France used to have relatively high ranking during 1999–2005, but due to lack of significant growth in the following years, it ranked sixth. In a similar fashion, Spain, regardless of sharp declines, was unable to catch up with other countries/regions and ranked seventh.

The remaining Turkey, Thailand and Greece are all BRI members that had somewhat similar development patterns during 1995–2006. In the following years, Turkey accelerated growth, which declined over 2014–2016 yielding to Thailand, but then regained its position back and ranked eighth in 2018. In its turn, Thailand, whose values were rather similar to those of Greece, broke away obtaining ninth ranking. Although Greece had positive trend, it was not enough to catch up and exceed other BRI countries/regions and, eventually, resulted in tenth ranking.

Comparing the in-strength of BRI and non-BRI groups, China again showed leadership owing to outstanding growth and now was accompanied by Poland. Next, the majority of countries/regions in both groups did not vary much (except China) and had somewhat similar trajectories. Although this time two BRI countries/regions had the highest rankings, Poland did not break away from other non-BRI countries and most of the BRI countries were again positioned below the non-BRI group.

### The underlying mechanism of the global tourism network

In our analysis, matrix of tourist flows was regressed by several matrices of independent variables that included physical distance, common language and religion, contiguity and economic distances for each year using the QAP method. Before conducting analysis, the matrices of tourist flows, physical and economic distances were transformed to logarithmic form. Table 5 presents the regression output over 1995–2018 in global, BRI and non-BRI tourist network.

**Table 5:** QAP regression results for selected years and average over 1995–2018.

	Year	Int.	Ph. dist.	Com. lang.	Contiguity	Com. rel.	Econ. dist.	Obs.	Adj. R^2^
**Global**	**1995**	0.004	-1.131***	0.862***	2.273***	0.813***	0.874***	45227	0.898
	**2005**	0.006	-1.211***	0.787***	2.430***	0.707***	0.894***	46864	0.884
	**2010**	0.008	-1.288***	0.790***	2.449***	0.247**	0.918***	46248	0.873
	**2015**	0.011	-1.357***	0.818***	2.373***	0.792***	0.949***	45533	0.871
	**2018**	0.011	-1.373***	0.621***	2.361***	0.704***	0.962***	46586	0.872
	**Avg.**	0.008	-1.236***	0.797***	2.434***	0.682***	0.903***	46016	0.879
**BRI**	**1995**	0.008	-1.135***	0.946***	2.852***	1.14***	0.854***	13865	0.873
	**2005**	0.010	-1.155***	1.323***	2.888***	0.535***	0.859***	14600	0.868
	**2010**	0.015	-1.260***	1.119***	2.803***	0.217	0.897***	14494	0.858
	**2015**	0.023	-1.355***	1.049***	2.972***	0.581***	0.939***	14396	0.848
	**2018**	0.023	-1.378***	0.860***	2.796***	0.733***	0.961***	14626	0.861
	**Avg.**	0.014	-1.199***	1.155***	2.918***	0.571*	0.877***	14362	0.861
**Non-BRI**	**1995**	0.002	-1.147***	0.549***	1.465***	0.09	0.919***	8826	0.924
	**2005**	0.005	-1.261***	0.188*	1.307***	0.677***	0.94***	8747	0.903
	**2010**	0.003	-1.354***	0.261***	1.462***	0.182	0.969***	8569	0.892
	**2015**	0.005	-1.345***	0.322***	1.086***	1.249***	0.967***	8325	0.897
	**2018**	0.004	-1.333***	0.144	1.218***	1.057***	0.961***	8823	0.891
	**Avg.**	0.004	-1.301***	0.269*	1.267***	0.644*	0.957***	8674	0.901

Significance levels

*** p < 0.001

** p < 0.01

* p < 0.05

From the adjusted R^2^ values, we can see that on average 87.9% of variance in the tourism network can be explained by physical distance, common language, contiguity, common religion and economic distance. Despite slight decline from 89.8% in 1995 to 87.2% in 2018, the value of R^2^ could explain the majority of variance in every year.

Speaking of independent variables, physical distance represented major obstacle for flows in tourism networks, which is also consistent with results obtained from gravity tourism demand models [28]. Judging by its coefficients, we can observe that physical distance had greater adverse effect on tourist flows in BRI countries/regions compared to non-BRI. Common language that reflects cultural similarity [29] exhibited significant positive influence and turned out to be more important for BRI tourists. Contiguity, used to measure geographic proximity [30], reported significant positive influence with the highest coefficients compared to those of other variables and had much greater impact on the BRI group. Common religion, which is another proxy of cultural similarity in gravity models [31], influenced tourist flows at 5% significance level in BRI and non-BRI samples with stronger impact on the latter one. Unexpectedly, economic distance that represents difference in economic development [49] between countries/regions demonstrated positive influence meaning that tourists were willing to travel to countries/regions, whose economic development was different from that of their home countries. This factor was more important for travelers from non-BRI group.

## Conclusions

### Discussion and conclusions

In this study, we investigated global tourist flows of 221 countries/regions over 1995–2018 using complex network analysis. The main goals of this research were to conduct descriptive analysis examining the evolution of structural properties of tourism network in the context of B&R Initiative and apply exploratory analysis to define on annual basis the underlying mechanisms of global tourism network as well as BRI and non-BRI tourism networks.

At macro level, the descriptive part indicated that global tourism network was a sparse network and exhibited small world properties. Having much fewer links than possible maximum, our network exhibits scale-free power-law distribution [44], whereas its actors are able to reach each other through a relatively small number of connections [45]. At the same time, growing values of network density over time might be related to the globalization trend [43] and government policies (such as the BRI) aimed to strengthen tourism exchanges [4]. These assumptions can be justified by declining betweenness centralities and increasing of particular countries/regions in global tourism network that show diminishing power of their intermediary roles along with growing degree and strength centralities that imply increasing connectivity between them.

At micro level, the fluctuations of out- and in-strength centralities in particular countries/regions could be related to events including severe acute respiratory disease (2003), global financial crisis (2008), the influenza A (H1N1) epidemic (2009), however their magnitude was rather smaller compared to regional crises. As an example, political instability in Ukraine (lasting since 2013) [47], following economic sanctions imposed on Russia (since 2014) [48], coup d’état in Thailand (2014) [50] and Russia’s temporary travel ban to Turkey (2015) had far more severe effects on tourism of the respective countries/regions affecting their out- and in-strength centralities.

Tourism literature suggests that factors influencing tourist flows might be related to tourists’ income [9], overall destination quality [28], demographic structure [51], government policies [52] and tourism attractiveness [53] among others. For instance, following unprecedented economic growth, Chinese inbound and outbound tourism saw considerable increase [46, 54] further enhanced by more recent Belt and Road Initiative [2, 5]. In a similar way, Germany and the USA have high out-strength centralities owing to developed economies and large populations. Unimpeded by political instability, Ukraine’s out-strength continued steady growth and was facilitated by visa liberalization with the European Union in 2017, which particularly was contributing to the in-strength centrality of Poland. A number of countries/regions with high in-strength were regarded attractive from tourists’ perspective as classic beach tourism destinations (e.g. Italy, Thailand) and/or having rich cultural and historical heritage (France).

Out-strength values showed that important BRI countries/regions in outbound tourism market included China, Russian Federation, Korea (Republic of), Ukraine and Saudi Arabia, while the non-BRI group was represented by Germany, Hong Kong (China), United Kingdom and France. There was a clear division between both groups, in which non-BRI was above BRI (except China) implying greater role of the former one in outbound tourism. As to inbound tourism market, the in-strength values reported that important BRI members were China, Poland, Greece, Thailand and Turkey, while non-BRI countries/regions included Italy, the United States, Hong Kong (China), France and Spain. In BRI group, China and Poland were followed by five non-BRI and the remaining three BRI countries/regions. Although the role of BRI countries/regions as attractors of inbound tourists was higher compared to the outbound tourism, the non-BRI group, in general, had more power.

In explanatory part, a more sophisticated analysis was conducted to verify whether gravity theory, a widely used framework in tourism modeling [32] that emphasizes the role of various dimensions of distance, would also have significant impact on tourist flows in network setting. To achieve this, the QAP methodology tested the impact of traditional gravity variables such as physical distance [28], common language [29], contiguity [30], common religion [31] and economic distance [49] in global, BRI and non-BRI tourism networks. Our results demonstrated that physical distance represented a major obstacle for the international tourism, while common language, contiguity and common religion diminished travel barriers between countries/regions increasing tourist flows. Unexpectedly, our models revealed that tourist flows rose with a greater economic distance between origin and destination countries/regions implying that different level of economic development plays role of facilitator in tourism networks. Factors such as common language and contiguity were more important in the BRI tourism network, while physical and economic distances as well as common religion played greater role in the non-BRI tourism network.

### Theoretical and managerial implications

Our study has several theoretical contributions. Firstly, we examine the nature of global tourist network and its development patterns in relation to major concepts of network theory. Whereas tourism studies related to BRI typically apply econometric modeling, our research is the first to apply complex network analysis to investigate the evolution of centrality characteristics of the BRI members and compare them to non-BRI group. In this regard, the tourism networks are investigated over a relatively long period, while the majority of publications with network analysis typically focuses on static networks [16, 17]. Finally, acknowledging that descriptive methodology cannot reveal the underlying mechanisms of network formation [15], our research employs a more sophisticated QAP analysis and verifies that gravity variables have significant influence on tourist flows in global, BRI and non-BRI networks.

The obtained findings imply a number of practical implications. Tourism in destination countries/regions should be developed considering existing and prospecting relationships with origin countries/regions. This means that future policies could prioritize important countries/regions while designing tourism strategies, developing tourism programs and constructing tourism facilities. At the same time, it is necessary to remember about tourists’ preferences such as inclination to visit neighboring countries/regions rather than distant ones, desire to travel to places with common language and religion as well as willingness to have travel experiences in economically different countries/regions. Lastly, policymakers should develop strategies by prioritizing the importance of each factor on tourists’ behavior in overall, BRI and non-BRI networks.

### Limitations and future research

In this study, the quality of tourism data led to the exclusion of 2019 year and influenced the development trends of structural properties in 2017 and 2018. By selecting tourism classifications with the largest data, our study could not precisely examine tourists’ behavior. In addition, a considerable number of countries/regions provide data in which tourist flows to a rather limited number of countries/destinations is available.

Future research might consider examining other network properties such as modularity, dyads, roles, page ranking and others. Community analysis can shed some light on how tourist flows between countries/regions in the network were evolving. Using the QAP, a number of political, social and weather factors could be further analyzed.

## Supporting information

S1 Table Summary of tourism studies related to the BRI. (PDF)

S2 Table List of the BRI countries/regions by the end of 2018. (PDF)
