# Supplementary material for: Global tourist flows under the Belt and Road Initiative: A complex network analysis
Source: PLoS One. 2022 Aug 16;17(8):e0272964. doi: 10.1371/journal.pone.0272964 (PMC9380932; doi:10.1371/journal.pone.0272964)
Supplement: S1 Table — (PDF) [file pone.0272964.s001.pdf]

S1 Table. Summary of tourism studies related to the BRI.

Literature	Research area	Methodology	Conclusions
Ahmad, Draz (20)	Tourism and environmental pollution	Fully modified ordinary least squares (1991-2016)	Tourism negatively affects environment in the majority of China's provinces, but has positive influence on environment in Xinjiang
Deng and Hu (2)	Spillover effects of China's outbound tourism	Spatial panel model (2006-2015)	Geographic and cultural proximities have significant effects on tourism flows, while geographic and cultural distances have negative impact
Huang, Han (5)	The influence of BRI policy on China's inbound tourism	Difference-in-differences (2008-2016)	The BRI policy is positively correlated with China's tourist generating markets
Li, Shi (4)	Impact of BRI policy on tourism economy	Difference-in-differences (2000-2017)	The BRI policy has positive influence on inbound tourist arrivals and inbound tourism revenue.
Liu and Suk (21)	Tourism between China and Azerbaijan	SWOT and Analytic Hierarchy Process	Tourism cooperation strategy should be based on internal strengths and external opportunities.
Li, Tavitiyaman (22)	Determinants of tourist arrivals from Mongolia and Russia	Multiple linear regression (1994-2017)	GDP, number of airlines and hotels positively influence tourist arrivals in China, while the number of Internet users have adverse effects.
Chen, Cui (23)	Drivers of international tourism development	Global vector autoregressive model (1995-2017)	China's economic growth has positive impact on the international tourism along the BRI and vice versa.
