# Supplementary material for: Global tourist flows under the Belt and Road Initiative: A complex network analysis
Source: PLoS One. 2022 Aug 16;17(8):e0272964. doi: 10.1371/journal.pone.0272964 (PMC9380932; doi:10.1371/journal.pone.0272964)
Supplement: S2 Table — (PDF) [file pone.0272964.s002.pdf]

S2 Table. List of the BRI countries/regions by the end of 2018.

Afghanistan	Ecuador	Malta	Sierra Leone
Albania	Egypt, Arab Rep.	Mauritania	Singapore
Algeria	El Salvador	Micronesia, Fed. Sts.	Slovak Republic
Angola	Estonia	Moldova	Slovenia
Antigua and Barbuda	Ethiopia	Mongolia	Somalia
Armenia	Fiji	Montenegro	South Africa
**Austria**	Gabon	Morocco	South Sudan
Azerbaijan	Gambia, The	Mozambique	Sri Lanka
Bahrain	Georgia	Myanmar	Sudan
Belarus	Ghana	Namibia	Suriname
**Benin**	Greece	Nepal	Tajikistan
Bolivia	Grenada	New Zealand	Tanzania
Bosnia and Herzegovina	Guinea	**Niger**	Thailand
Brunei Darussalam	Guyana	Nigeria	Timor-Leste
Bulgaria	Hungary	Niue	Togo
Burundi	Indonesia	North Macedonia	Tonga
Cabo Verde	Iran, Islamic Rep.	Oman	Trinidad and Tobago
Cambodia	Iraq	Pakistan	Tunisia
Cameroon	Kazakhstan	Panama	Turkey
Chad	Kenya	Papua New Guinea	Uganda
Chile	Korea, Rep.	Philippines	Ukraine
China (Mainland)	Kuwait	Poland	United Arab Emirates
Cook Islands	Kyrgyz Republic	Portugal	Uruguay
**Comoros**	Lao PDR	Romania	Uzbekistan
**Congo, Rep.**	Latvia	**Russian Federation**	Vanuatu
Costa Rica	Lebanon	Rwanda	Venezuela, RB
Côte d'Ivoire	Libya	Samoa	Vietnam
Croatia	Lithuania	Saudi Arabia	Yemen, Rep.
Czech Republic	Madagascar	Senegal	Zambia
Djibouti	Malaysia	Serbia	Zimbabwe
**Dominica**	Maldives	Seychelles	
Countries in **bold** had no BRI signing date, and are regarded as BRI in 2018			
